# Early-Onset Neutropenia Induced by Rituximab in a Patient with Lupus Nephritis and Hemolytic Anemia

**DOI:** 10.1155/2015/616787

**Published:** 2015-02-12

**Authors:** Mariangelí Arroyo-Ávila, Ruth M. Fred-Jiménez, Luis M. Vilá

**Affiliations:** Division of Rheumatology, Department of Medicine, University of Puerto Rico, Medical Sciences Campus, San Juan, PR 00936, USA

## Abstract

Rituximab is an anti-CD20 monoclonal antibody that has been used to treat several complications of systemic lupus erythematosus (SLE) including nephritis, cerebritis, and hematological disorders. Neutropenia is among the adverse events associated with rituximab; this usually occurs several weeks after therapy. However, early-onset neutropenia has been reported only in a few cases. Herein, we describe a 36-year-old Hispanic SLE woman who developed severe early-onset neutropenia (0.3 × 10^9^/L) after the second weekly rituximab infusion (375 mg/m^2^ weekly × 4) given for nephritis and hemolytic anemia. She also had early-onset thrombocytopenia after rituximab therapy. Both hematological disorders resolved 12 days after the fourth and final dose. This case, together with few others, suggests that early-onset neutropenia may occur during rituximab therapy. Even though rituximab-induced neutropenia seems to be transient, it may predispose SLE patients to severe complications such as infections.

## 1. Introduction

Rituximab is a chimeric monoclonal antibody directed against CD20 positive B cells. It was initially approved for the treatment of non-Hodgkin's lymphoma and later indicated for autoimmune rheumatic diseases such as rheumatoid arthritis and antineutrophil cytoplasmic antibody-associated vasculitis [1–3]. Furthermore, rituximab has been used as a second-line treatment for systemic lupus erythematosus (SLE), especially in refractory nephritis, cerebritis, and hematological disorders [1, 4–6]. In general, rituximab has a favorable safety profile; however, several adverse events have been described. Among the most commonly reported are infusion reactions, infections, and late-onset neutropenia [3–5, 7, 8]. The latter occurs after 4 weeks of rituximab treatment, with a median time to development reported between 14 and 23 weeks in patients with rheumatic disorders [7, 9]. Early-onset neutropenia during rituximab infusion is rare [8–11]. Herein, we report an SLE patient who developed severe early-onset neutropenia during treatment with rituximab given for lupus nephritis and hemolytic anemia.

## 2. Case Presentation

A 36-year-old Puerto Rican woman was hospitalized to our institution in March 2010 because of nephrotic syndrome and hemolytic anemia. Seven years before admission, she was diagnosed with SLE manifested by constitutional symptoms, malar rash, photosensitivity, oral ulcers, alopecia, Raynaud's phenomenon, arthritis, myalgias, lymphadenopathy, lymphopenia, anemia, positive antinuclear antibodies (ANA), elevated anti-dsDNA antibodies, and hypocomplementemia (C3 and C4). Early during the course of disease, she developed autoimmune pancreatitis, serositis, and membranous glomerulonephritis. Initially, she was treated with corticosteroids (including intravenous [IV] methylprednisolone therapy), hydroxychloroquine, IV pulse cyclophosphamide, and azathioprine. However, she had either partial clinical response or adverse events to these drugs for which she was started on mycophenolate mofetil in 2007. She achieved good clinical response with this therapy.

One month before admission, mycophenolate mofetil was discontinued due to severe diarrhea resulting in a severe SLE exacerbation. Upon admission she had oral ulcers, an erythematous maculopapular rash on her face, neck, and upper extremities, and anasarca. Initial laboratories showed a white blood cell (WBC) count of 6.6 × 10^9^/L, neutrophil count of 6.3 × 10^9^/L, lymphocyte count of 1.3 × 10^9^/L, platelet count of 100 × 10^9^/L, and hemoglobin of 8.7 g/dL. The peripheral blood smear revealed slight hypochromasia, anisocytosis, and poikilocytosis and the presence of reticulocytes. No schistocytes were seen. The reticulocyte count was elevated at 2.6% and the haptoglobin was decreased at 5.8 mg/dL. She had a serum creatinine of 1.53 mg/dL that rapidly worsened to 2.29 mg/dL after 48 hours. Serum albumin was decreased at 1.8 g/dL. The urinalysis showed >50 red blood cells (RBC)/high power field (hpf), 0–4 leukocytes/hpf, 3+ protein, and 0–2 granular casts/hpf. The urine protein-to-creatinine ratio was 2.1 which worsened to 8.6 eleven days later. The Westergren sedimentation rate was elevated at 48 mm/hr. Complement C3 and C4 were decreased at 14.6 mg/dL (90–180 mg/dL) and 2.0 mg/dL (10–40 mg/dL), respectively. Anti-dsDNA antibodies were not elevated.

**Figure 1:**
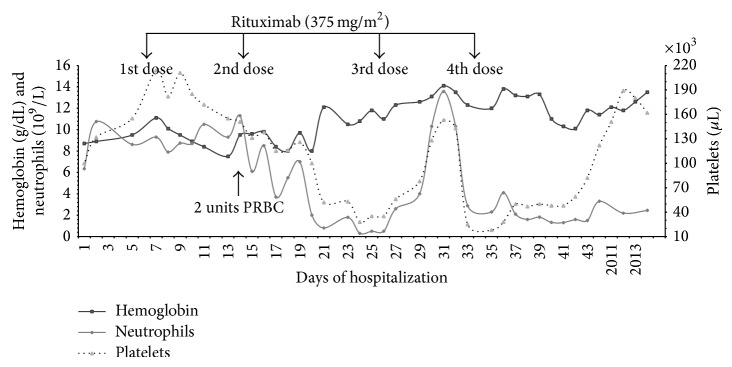
Hemoglobin, neutrophil count, and platelet count during hospitalization. Marked decreases in neutrophils and platelets are observed after seven days of the second rituximab infusion. Hemoglobin levels increased eight days after the second rituximab infusion and remained stable throughout hospitalization. Cell count returned to normal levels twelve days after the final rituximab dose. After four years of follow-up, hemoglobin, neutrophil count, and platelet count remained normal.

The patient was treated with methylprednisolone 250 mg IV every six hours for 3 days, followed by methylprednisolone 1-2 mg/kg/day. Mycophenolate mofetil was restarted. During her hospital course she developed several complications including seizures, oliguria requiring transient hemodialysis, hypertensive crisis, and ascites. Rituximab 675 mg (375 mg/m^2^) IV weekly was started. Fifteen days after the first rituximab dose (7 days after the second infusion), the WBC count decreased to 2.8 × 10^9^/L, neutrophil count to 2.0 × 10^9^/L, and lymphocyte count to 0.6 × 10^9^/L (Figure 1). The next day, platelets decreased to 52 × 10^9^/L. Cell counts continued to decrease with a nadir of 0.8 × 10^9^/L for leukocytes, 0.3 × 10^9^/L for neutrophils, and 28 × 10^9^/L for platelets, 19 days after the first rituximab dose. Conversely, hemoglobin levels increased during this time. Thirty-nine days after the first rituximab dose (12 days after the fourth and final infusion), WBC and platelet counts returned to normal limits.

She responded favorably to rituximab therapy. Renal function improved and proteinuria significantly decreased. Upon discharge she had a WBC count of 4.6 × 10^9^/L, neutrophil count of 3.3 × 10^9^/L, platelet count of 122 × 10^9^/L, hemoglobin of 11.4 g/dL, and serum creatinine of 0.9 mg/dL. She was discharged on prednisone 60 mg daily, hydroxychloroquine 200 mg twice daily, and mycophenolate mofetil 500 mg twice daily. Prednisone dose was gradually decreased until discontinued. After 4 years of follow-up the patient remained in clinical remission with normal WBC and platelet counts and renal function.

## 3. Discussion

We describe a patient with SLE manifested by nephritis and hemolytic anemia who developed early-onset neutropenia after the second weekly infusion of rituximab. Early-onset neutropenia, occurring earlier than 4 weeks after initiation of rituximab therapy, has been rarely reported in SLE. To our knowledge, three additional cases have been described (Table 1) [8, 11]. Enríquez et al. reported a 48-year-old woman with SLE and diffuse proliferative glomerulonephritis refractory to cyclophosphamide and high-dose corticosteroids who developed severe neutropenia 5 days after the second dose of rituximab [11]. Additionally, Gottenberg et al. described 2 patients with resistant SLE treated with rituximab after failing several immunosuppressive therapies including cyclophosphamide [8]. Both patients developed severe neutropenia in less than 15 days after the first rituximab infusion. One of these 2 patients, as well as our case, was concomitantly taking mycophenolate mofetil. In the LUNAR trial, Rovin et al. reported that patients receiving rituximab concurrently with mycophenolate mofetil (1.5 gm/day–3 gm/day) for lupus nephritis had a higher frequency of neutropenia compared with those taking mycophenolate mofetil alone (2.7% versus 1.4%). The severity or degree of neutropenia was not reported [4].

The differential diagnosis of transient neutropenia in our patient includes lupus itself, infections, and other drugs besides rituximab, but these possibilities seem unlikely. The patient had mild leukopenia in the past. However, the lowest leukocyte count was 3.0 × 10^9^/L which was documented 3 months prior to current hospitalization. She did not present clinical findings of viral or bacterial infection and had negative blood cultures during the period of rituximab treatment. Other than rituximab, she did not receive drugs that could potentially induce neutropenia.

In addition to neutropenia, early-onset thrombocytopenia may occur following rituximab therapy [10, 12, 13]. Our patient developed transient thrombocytopenia after the second rituximab infusion. Likewise, Larrar et al. reported a two-year-old boy with resistant autoimmune hemolytic anemia who developed uncomplicated transient thrombocytopenia 1 week after the third dose of rituximab [10]. He had spontaneous recovery of platelet count 7 days later. Similar abnormalities have been reported in patients with hairy cell leukemia and mantle cell lymphoma [12, 13]. To our knowledge, no cases of early-onset thrombocytopenia, aside from ours, have been reported in SLE patients receiving rituximab treatment.

Rituximab-induced cytopenias, either early or late, appear to be transient and self-limited [7–11]. While some authors report successful therapy with granulocyte-colony stimulating factor [10, 11], others note recovery of cell counts after discontinuation of rituximab alone [7–9]. Rituximab-induced neutropenia may cause neutropenic fever, infections, and sepsis [5, 7–9, 11]. Our patient had complete resolution of hematological abnormalities after rituximab discontinuation and she did not develop any complications related to neutropenia. Interestingly, our patient had an increase of neutrophil and platelet counts between rituximab doses 3 and 4. This rebound was probably related to the effect of corticosteroid therapy. As described before, during this period the patient was receiving high-dose intravenous methylprednisolone but this was increased from 80 mg daily to 120 mg daily 1 day prior to the rebound.

The pathophysiology of rituximab-induced cytopenia remains unclear as neutrophils and platelets do not express CD20. Using direct immunofluorescence testing, Voog et al. described anti-neutrophil IgG antibodies bound to the surface of neutrophils in two lymphoma patients treated with rituximab who developed neutropenia. However, serum anti-neutrophil IgG antibodies were not detected in these patients [14]. Weissmann-Brenner et al. found that plasma from a lymphoma patient who developed neutropenia after rituximab completely inhibited granulocyte growth of a healthy bone marrow [15]. Nevertheless, most proposed mechanisms attempt to explain the development of late-onset neutropenia, which is more commonly seen. Early-onset neutropenia following rituximab administration may occur through different mechanisms that have yet to be elucidated.

In summary, we report a Hispanic woman with SLE who presented with early-onset neutropenia after the second weekly infusion of rituximab. She also had transient thrombocytopenia. Although cytopenias are common in SLE, in our patient they do not seem to be related with active disease given that other activity parameters such as anemia, proteinuria, and renal insufficiency were resolving. Hemoglobin levels began to increase 16 days after the first rituximab infusion (coinciding with neutrophil decrease) and remained stable thereafter. In addition, there was a sequential association between rituximab administration and the development of cytopenias. Although early-onset cytopenias have been rarely reported, they may be underestimated since follow-up blood cell counts are not routinely performed after rituximab infusions. While rituximab-induced cytopenias seem to be transient they may predispose SLE patients to more severe complications such as infections and sepsis. Clinicians should be aware of the possibility of early-onset neutropenia and thrombocytopenia following rituximab treatment. Repeated clinical assessments to evaluate cytopenia-related complications are reasonable and may be used to guide therapy.

## Figures and Tables

**Table 1:** Adult SLE patients presenting with early-onset neutropenia associated with rituximab therapy.

Author/year	Gender/age (years)/ethnicity	Active clinical manifestations	Prior immunosuppressive therapy	Concomitant immunosuppressive treatment	Number of weekly rituximab infusions/dose	Time to neutropenia after 1st dose of rituximab (days)
Gottenberg et al./2005 [8]	F/30/NA	Pleuropericarditis	NA	None	1/375 mg/m^2^	10
Gottenberg et al./2005 [8]	F/22/NA	Articular	Cyclophosphamide	Mycophenolate mofetil	4/375 mg/m^2^	15
Enríquez et al./2007 [11]	F/48/Caucasian	Polyarthritis, nonnephrotic range proteinuria	Cyclophosphamide	None	2/375 mg/m^2^	15
Current report/2014	F/32/Hispanic	Oral ulcers, rash, hemolytic anemia, and nephrotic syndrome	Cyclophosphamide	Mycophenolate mofetil	4/375 mg/m^2^	15

SLE: systemic lupus erythematosus; NA: not available.
